# SCoT: a Python toolbox for EEG source connectivity

**DOI:** 10.3389/fninf.2014.00022

**Published:** 2014-03-11

**Authors:** Martin Billinger, Clemens Brunner, Gernot R. Müller-Putz

**Affiliations:** Institute for Knowledge Discovery, Graz University of TechnologyGraz, Austria

**Keywords:** electroencephalogram, connectivity, Python, single-trial, brain-computer interface

## Abstract

Analysis of brain connectivity has become an important research tool in neuroscience. Connectivity can be estimated between cortical sources reconstructed from the electroencephalogram (EEG). Such analysis often relies on trial averaging to obtain reliable results. However, some applications such as brain-computer interfaces (BCIs) require single-trial estimation methods. In this paper, we present SCoT—a source connectivity toolbox for Python. This toolbox implements routines for blind source decomposition and connectivity estimation with the MVARICA approach. Additionally, a novel extension called CSPVARICA is available for labeled data. SCoT estimates connectivity from various spectral measures relying on vector autoregressive (VAR) models. Optionally, these VAR models can be regularized to facilitate ill posed applications such as single-trial fitting. We demonstrate basic usage of SCoT on motor imagery (MI) data. Furthermore, we show simulation results of utilizing SCoT for feature extraction in a BCI application. These results indicate that CSPVARICA and correct regularization can significantly improve MI classification. While SCoT was mainly designed for application in BCIs, it contains useful tools for other areas of neuroscience. SCoT is a software package that (1) brings combined source decomposition and connectivtiy estimation to the open Python platform, and (2) offers tools for single-trial connectivity estimation. The source code is released under the MIT license and is available online at github.com/SCoT-dev/SCoT.

## 1. Introduction

Quantifying interactions between brain areas is an important and useful tool in neuroscience (Michel and Murray, 2012). Spatially separated brain areas form dynamic large-scale networks that are described by functional and effective connectivity (Schnitzler and Gross, 2005; Siegel et al., 2012). While functional connectivity measures synchronous activation, effective connectivity explains causal relations between areas (Friston, 1994, 2011). We will use the term connectivity for both functional and effective connectivity throughout this manuscript.

Estimates of connectivity can be deduced from the multi-channel EEG by employing a VAR model. However, fitting such a model requires a large amount of data. In particular, the required number of time samples is proportional to the number of channels and the model order. A common approach to generate enough data is to use repeated trials of the same task. However, the EEG also contains task-related activity that varies from trial to trial, which would disappear when averaging over trials. Such activity can only be studied at the single-trial level (Michel and Murray, 2012). A framework for performing single-trial time-varying system identification and visualization was published recently (Mullen et al., 2013). An important use case for single-trial analysis are BCIs, which extract control signals from ongoing brain activity (Millán et al., 2010). Connectivity measures have already been used in several BCI-related studies (Gysels et al., 2005; Shoker et al., 2005; Brunner et al., 2006; Wei et al., 2007; Hamner et al., 2011; Lim et al., 2011; Daly et al., 2012; Billinger et al., 2013a).

Measuring connectivity from the EEG entails methodological challenges such as volume conduction and multiple comparison problems (Siegel et al., 2012). Due to volume conduction, electrical signals originating from one source in the brain are detected by multiple EEG electrodes. Conversely, each electrode measures a superposition of activity from multiple sources. Thus, interpreting connectivity between EEG channels if of limited usefulness. This can be overcome by transforming the problem from the electrode domain to the source domain. Common approaches to estimate source activities include source localization techniques and independent component analysis (ICA). Source localization attempts to map the scalp potential distribution to current source densities on the cortex. However, this approach requires accurate models of head anatomy and electrical properties, as well as accurate electrode locations (Baillet et al., 2001). In contrast, ICA performs a blind decomposition of EEG channels without having to rely on a head model. Additionally, source signals obtained from ICA can be interpreted as originating from cortical dipoles (Makeig et al., 1996). When measuring connectivity between ICA sources, the seemingly contradictory assumptions of dependence for connectivity estimation and independence for ICA must be carefully taken into account. We give a detailed discussion of this issue in section 2.

Our source connectivity toolbox (short SCoT) is a software package for Python that contains tools for estimating connectivity between cortical sources. While various implementations of connectivity are available on other platforms, source connectivity toolbox (SCoT) is the first Python package dedicated to connectivity estimation. Apart from common multi-trial analysis techniques, SCoT also supports single-trial connectivity. The toolbox contains separate modules for ICA, VAR model fitting, and spectral connectivity measure estimation (supported measures are listed in Table 1). While the tools were originally designed for single-trial BCI feature extraction, they equally work with multiple trials and are useful for functional and effective connectivity analysis of EEG signals. SCoT implements the MVARICA approach (Gómez-Herrero et al., 2008), which combines VAR models and ICA for jointly estimating sources and connectivity. Furthermore, we implemented a novel supervised variant of MVARICA specifically tailored toward classification of BCI data, which we named CSPVARICA. The toolbox contains built-in routines for data processing, but can be configured to use the machine learning package scikit-learn (Pedregosa et al., 2011) or custom routines as backends. Unit tests assure correct functionality of the core routines.

**Table 1:** **VAR-derived measures included in SCoT**.

**Measure**	**Description**
A	Spectral representation of the VAR coefficients
H	Transfer function that transforms the innovation process into the VAR process
S	Cross spectral density
G	Inverse cross-spectral density
PHI	Phase angle
COH	Coherence Nunez et al., 1997
pCOH	Partial coherence Franaszczuk et al., 1985
PDC	Partial directed coherence Baccalá and Sameshima, 2001
ffPDC	Full frequency partial directed coherence
PDCF	PDC factor Baccalá and Sameshima, 2001
GPDC	Generalized partial directed coherence Faes et al., 2012
DTF	Directed transfer function Kamiński and Blinowska, 1991
ffDTF	Full frequency directed transfer function Korzeniewska et al., 2003
dDTF	Direct directed transfer function Korzeniewska et al., 2003
GDTF	Generalized directed transfer function (known as directed coherence) Faes et al., 2012

The aim of this article is twofold. First, we want to introduce SCoT to researchers along with code snippets that show basic usage examples. Second, we give a technical overview of the methods we implemented and present our new CSPVARICA.

## 2. Materials and methods

### 2.1. Source estimation and VAR model fitting

The EEG is commonly modeled as a linear mixture of underlying cortical source activations (1). These source activations are modeled as VAR processes (2), which contain information about connectivity (Gómez-Herrero et al., 2008).

(1) $${\text{x}}_{n}={\text{Ms}}_{n}$$

(2) $${\text{s}}_{n}=\sum_{k=\;1}^{p}\;{\text{B}}^{\left(k\right)}{\text{s}}_{n-k}+{\text{e}}_{n}$$

The mixing matrix **M** transforms every sample *n* of the source activations **s**_*n*_ into the observable EEG, which is denoted as **x**_*n*_. VAR model coefficient matrices **B**^(*k*)^ and innovation process **e**_*n*_ form the VAR model of order *p* that describes the source activations; **e**_*n*_ is assumed to be a vector of independent non-Gaussian white noise processes.

The most naive approach to connectivity estimation is to ignore the mixing of cortical sources and assume that each EEG sensor corresponds to a unique cortical source. However, volume conduction between EEG sensors is not correctly captured by VAR models and therefore severely limits the usefulness of connectivity at the sensor level (Siegel et al., 2012).

**Figure 1:**
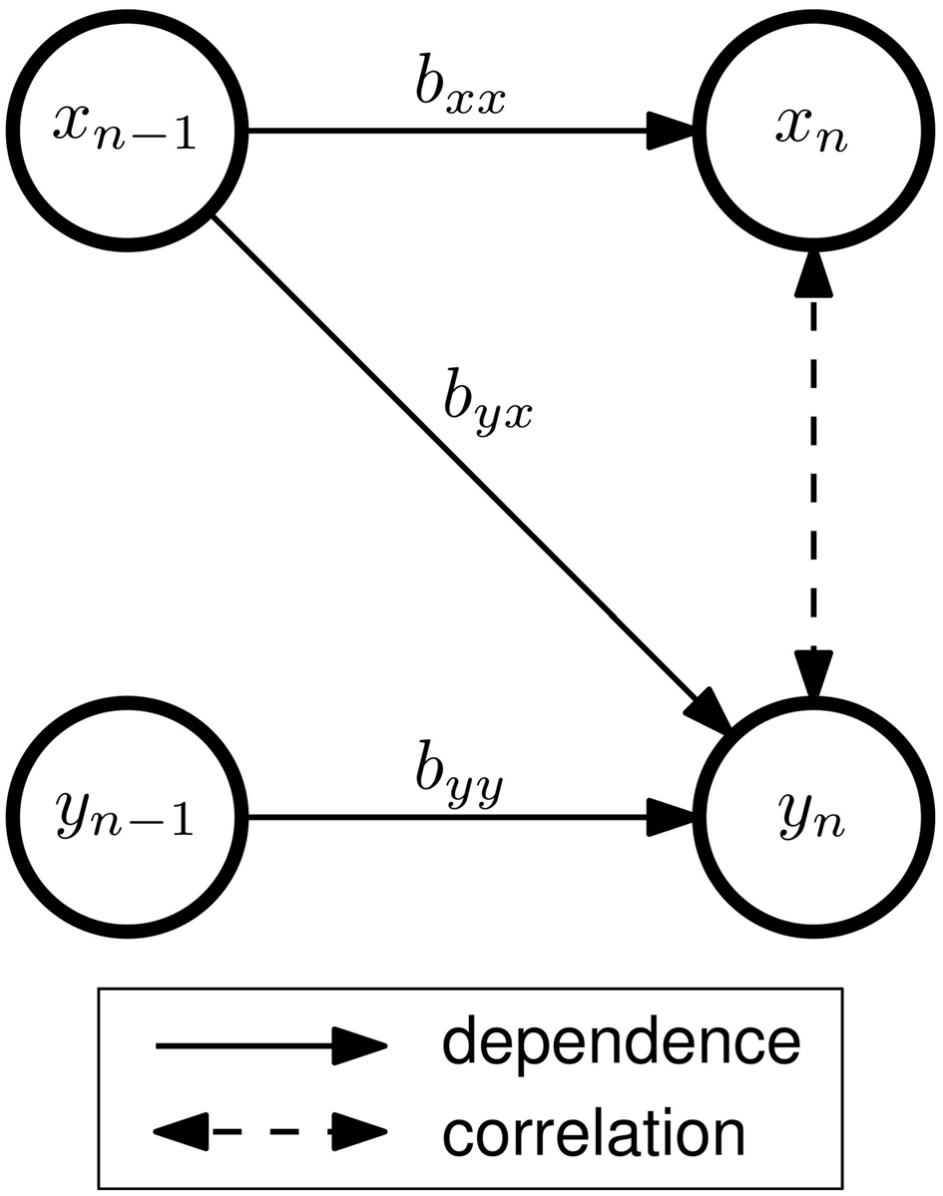
**Connectivity and correlation in a bivariate AR(1) model of order *p* = 1 with two signals *x* and *y***. The VAR coefficients *b*_*ij*_ describe the dependency of signal *i* on the previous sample of signal *j*. In this example, there is effective connectivity from *x* to *y*, but not from *y* to *x*. Although no direct interaction between *x*_*n*_ and *y*_*n*_ exists, the signals are correlated, since both *x*_*n*_ and *y*_*n*_ depend on *x*_*n*−1_. The strength of this correlation depends on *b*_*yy*_, *b*_*yx*_, and additive noise (which is not shown in this figure).

More sophisticated approaches obtain source activations by constructing an unmixing matrix **U** that mathematically reverses the mixing process so that **s**_*n*_ = **Ux**_*n*_ (with **UM** equal to the identity matrix **I**). ICA performs such a decomposition by attempting to find spatial EEG components with minimal instantaneous cross-dependencies between each other. These components can be interpreted as cortical source activations that are not affected by volume conduction. However, connectivity between sources can also cause instantaneous cross-dependencies (Figure 1). ICA assumes no temporal dependence structure within the data, but connectivity between sources violates this assumption. Thus, ICA treats the signals as though instantaneous cross-dependencies were exclusively caused by the mixing process. This might decrease the reliability of subsequent connectivity estimates, as was demonstrated by Haufe et al. (2010).

We provide an implementation of ICA source decomposition in SCoT. However, limitations and conflicting assumptions of VAR and ICA should be carefully considered before applying this approach. Therefore, we recommend more sophisticated techniques such as MVARICA or our novel CSPVARICA.

### 2.2. MVARICA

**Figure 2:**
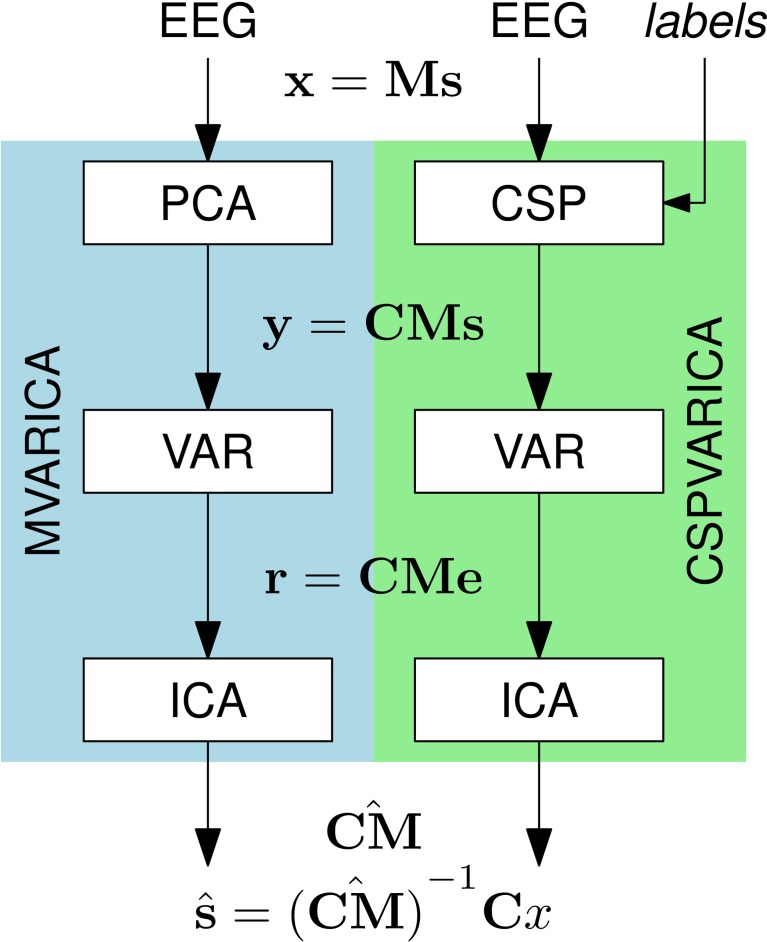
**Source decomposition with MVARICA and CSPVARICA**. Both approaches estimate source activations ŝ from the EEG. The EEG signals x are transformed to y either by PCA (MVARICA) or CSP (CSPVARICA). Subsequently, the residuals r of a VAR model fitted to y are decomposed by ICA to obtain an estimate of the combined matrix CM. This matrix, along with the transformation C is used to obtain estimated source activations ŝ.

SCoT implements the MVARICA approach, which performs joint source decomposition and VAR model fitting while respecting their respective assumptions about dependencies (Gómez-Herrero et al., 2008). MVARICA works in three steps (see Figure 2). First, the EEG is transformed by applying principal component analysis (PCA) as follows:

(3) $${\text{y}}_{n}={\text{Cx}}_{n}={\text{CMs}}_{n}$$

The signals in **y**_*n*_ contain the PCA-transformed EEG. The PCA transformation matrix **C** is pruned to remove components that contribute least to the total EEG variance. This step reduces the dimensionality for subsequent processing and limits the number of sources found by MVARICA. Second, a VAR model with coefficients **A**^(*k*)^ and residual processes **r**_*n*_ is fitted to **y**_*n*_:

(4) $${\text{y}}_{n}=\sum_{k=\;1}^{p}\;{\text{A}}^{\left(k\right)}{\text{y}}_{n-k}+{\text{r}}_{n}$$

By combining (2–4) we can relate the VAR model fitted to **y**_*n*_ with the VAR model that describes the source activations:

(5) $${\text{A}}^{\left(k\right)}=(\text{CM}){\text{B}}^{\left(k\right)}{\left(\text{CM}\right)}^{-1}$$

(6) $${\text{ r}}_{n}=(\text{CM}){\text{e}}_{n}$$

The residuals **r** contain cross-dependencies that cannot be explained by the VAR model. According to (6), all cross-dependencies remaining in the residuals are due to the transformation **CM**. In the third step, the residuals are decomposed by ICA in order to obtain an estimate of the transformation $\hat{\text{CM}}$. Finally, an estimated unmixing matrix $\hat{\text{U}}$ is obtained as $\hat{\text{U}}={\left(\hat{\text{CM}}\right)}^{-1}\text{C}$ so that

(7) $${\hat{\text{s}}}_{n}=\hat{\text{U}}{\text{x}}_{n}.$$

Typically, estimates of the VAR coefficients at the source level are obtained by inserting estimates $\hat{\text{A}}$ and $\hat{\text{CM}}$ in (5) and solving for $\hat{\text{B}}$. However, if sources are spatially stationary (constant **M**), the unmixing estimate $\hat{\text{U}}$ can be reused on other data sets to obtain source activations and fit VAR models directly to these activations according to (2). This allows us to determine the unmixing matrix from a set of training data, and subsequently perform connectivity estimation between the same sources on new data.

In general, MVARICA is applied to multi-trial data. Different strategies to obtain the VAR residuals **r** can be employed, depending on which stationarity assumptions hold on the data. If stationarity can be assumed across all trials, a single VAR model may be fitted to all trials. If stationarity can be assumed only across trials from the same condition, a different VAR model may be fitted for each condition. Finally, if stationarity cannot be assumed across trials, a different VAR model may be fitted for each individual trial. The last approach requires single-trial VAR model fitting, which we will discuss in section 2.6.

### 2.3. CSPVARICA

MVARICA reduces the input dimensionality by discarding the principal components that contribute least to the total EEG variance. However, EEG components of interest often have a low signal-to-noise ratio. Thus, PCA might remove such components while retaining noise with higher variance. We propose to use common spatial patterns (CSP) instead of PCA. While PCA finds components according to their contribution to the total variance, CSP finds components that explain the differences (in variance) between two conditions (Koles et al., 1990). Thus, we expect CSP to be superior to PCA whenever differences between two conditions (e.g., baseline/task, task/task, etc.) are analyzed. CSPVARICA is implemented in SCoT as a supervised alternative to MVARICA (see Figure 2). CSPVARICA is similar to MVARICA except for the first step, where we replaced PCA with CSP. Equations (3–7) are equally valid for CSPVARICA; only the tranformation matrix **C** is different because it represents the CSP transform instead of the PCA transform.

### 2.4. Model validation

MVARICA and CSPVARICA apply ICA to the residuals of VAR models. ICA assumes no temporal structure in the data. Thus, it is important that the VAR models adequately describe the data so that the residuals are serially uncorrelated.

SCoT implements a multivariate portmanteau test to test for whiteness in the residuals (Hosking, 1980). The test compares the multivariate Li-McLeod statistic *Q* against the null hypothesis that the residuals are white (Lütkepohl, 2005). When the residuals are normally distributed, *Q* follows a chi-squared distribution. However, we explicitly assume non-Gaussian residuals. Thus, we estimate the distribution of *Q*_0_ under the null hypothesis from surrogate residuals. Temporal structure in the residuals is destroyed by randomly permuting the residuals along the time axis. We repeatedly calculate *Q*_0_ from different permutations and calculate the probability of observing a *Q*_0_ larger than *Q*. A probability of less than 0.05 indicates significantly non-white residuals.

### 2.5. Connectivity

Once source estimates are available, numerous connectivity measures can be extracted from VAR models. Table 1 lists all measures currently implemented in SCoT. Some of the most commonly used measures are summarized in Schlögl and Supp (2006). All these measures may be extracted directly from the VAR coefficients returned by MVARICA or CSPVARICA.

In addition to well known measures, SCoT implements the full frequency partial directed coherence (ffPDC). The ffPDC is obtained by normalizing the partial directed coherence (PDC) over all frequencies instead of each frequency individually:

(8) $${\text{ffPDC}}_{ij}(z)=\frac{\left|A_{ij}(z)\right|}{\sqrt{\sum_{z}A_{:j}^{H}(z)A_{:j}(z)}}$$

Here, *i* and *j* correspond to the indices of the sink and the source signals, respectively. **A** is the inverse of the VAR transfer function.

In general, repeatedly applying MVARICA or CSPVARICA to different data segments yields components in different order. This makes tracking of connectivity patterns difficult. However, it is possible to overcome this issue by re-using the unmixing matrix obtained from one decomposition. Applying this unmixing matrix to new portions of data results in varying activations of the same sources, which facilitates three important use cases: single-trial estimation, time-varying analysis, and comparing different conditions.

### 2.6. Single-trial estimation

VAR model fitting typically requires a large amount of data. To obtain a sufficient amount of data, a model can be fitted to multiple trials, assuming stationarity across trials (i.e., each trial is a realization of the same process). However, this is not feasible in applications such as MI BCIs, where trial duration is several seconds or continuous control is required. Instead, connectivity estimates need to be obtained from a single window of data. In SCoT, we perform single-trial connectivity estimation on short windows of source activations, but use a training set of multiple trials to obtain sources that are assumed to be spatially stationary.

One procedure for single-trial connectivity estimation is described in our previous work (Billinger et al., 2013a), where various connectivity measures were estimated on selected ICA sources. This procedure can be improved by replacing ICA and source selection with MVARICA or CSPVARICA. Importantly, single-trial VAR model fitting is prone to overfitting due to the limited amount of data available in a single trial. However, this problem can be alleviated by regularization. Therefore, SCoT supports ridge regression for fitting VAR models to individual and multiple time windows. The scikit-learn backend provides additional model fitting routines including Lasso, Elastic Net, and generalized cross-validation (GCV) for determining the ridge parameter.

The degrees of freedom when fitting a VAR model depends on the model order. Typically, the order of VAR models is determined with cross-validation or selection criteria such as Akaikein formation criterion (AIC) or Bayesian information criterion (BIC). Regularization effectively limits the degrees of freedom, so both model order and regularization penalty can be optimized. For simplicity, we manually set the model order to a reasonably high value and optimize only the regularization penalty.

### 2.7. Statistics

So far, we have only discussed point estimates of connectivity. However, scientists will usually want to make statistical inferences about connectivity to either determine if there really is connectivity from one source to another, or to determine if there is a difference in connectivity between two conditions.

In SCoT, presence of connectivity is deduced using the method of surrogate data generated by phase randomization. While removing connectivity from each signal pair individually may give better results (Faes et al., 2009), we took a simpler approach, where connectivity is destroyed between all signals simultaneously. We obtain the distribution of connectivity under the null hypothesis of no connectivity by repeatedly estimating connectivity on surrogates. Connectivity estimated from actual data can be compared against this distribution to test if it is significantly non-zero.

Statistical difference in connectivity is obtained by performing bootstrap resampling. Bootstrap samples are drawn at the trial level, thus this method only works for multi-trial data sets. The distribution of difference in connectivity is obtained by bootstrapping both conditions. The difference is significantly different from zero if the confidence interval does not contain 0.

Typically, such tests are performed for each frequency bin and channel pair, which requires correction for multiple testing. However, individual tests are likely to be positively correlated. Thus, controlling for the family-wise error rate is likely to be overly conservative. Instead, controlling for the false discovery rate (Benjamini and Hochberg, 1995) is implemented in SCoT.

### 2.8. Source connectivity workflow

SCoT implements routines for estimating connectivity between EEG sources. Two estimation approaches are possible in SCoT. Too many Ps in approach is to estimate sources and connectivity jointly on the same data set using MVARICA or CSPVARICA. Alternatively, a two-step approach is supported, where sources and connectivity are estimated on different data sets. Both approaches impose strong spatial stationarity assumptions on the sources. However, in the latter approach source activity may vary over time.

Joint estimation is performed by applying MVARICA or CSPVARICA to a data set where sources are spatially and temporally stationary. Two-step estimation consists of separately performing source decomposition and VAR model fitting, possibly on different data sets. MVARICA or CSPVARICA can be employed in the source decomposition step by discarding their VAR estimates. In the second step, the unmixing matrix is used to obtain source activations on a different data set. Connectivity measures are estimated from VAR models fitted to these source activations.

The two-step approach is useful whenever connectivity is expected to vary between spatially stationary sources. Possible applications include comparing different conditions, analyzing time-varying connectivity, and estimating single-trial connectivity.

It is often useful to estimate time-varying connectivity. This can be done on a multi-trial or a single-trial basis, whichever is appropriate for the data and the research question. Time-varying multi-trial connectivity estimation facilitates analysis of cue-locked connectivity dynamics. Here, we estimate connectivity on the first time segment of multiple trials, then for the second (possibly overlapping) segment, and so on. This results in an average time-course of connectivity related to the trial start. Time-varying single-trial connectivity estimation may be employed when connectivity dynamics are not cue locked, or no cues are available such as in continuous BCIs. Here, we estimate connectivity on short (possibly overlapping) windows, resulting in a time course of instantaneous connectivity.

## 3. Implementation

### 3.1. Overview

**Figure 3:**
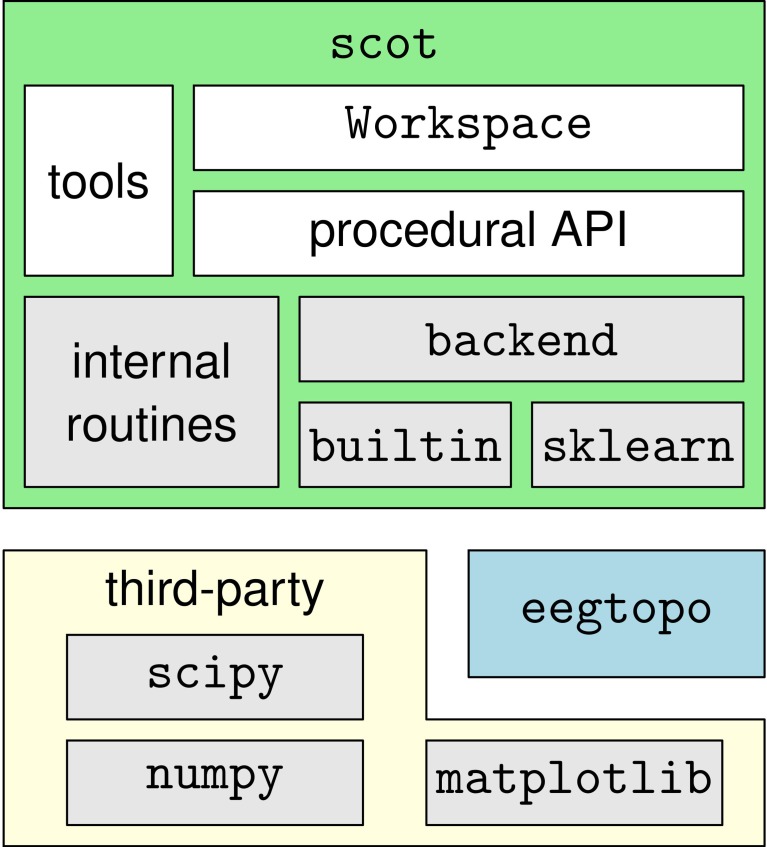
**Overview of the dependency hierarchy in the SCoT API**. High-level interfaces are depicted above the interfaces they depend on.

SCoT is distributed as a Python package named scot. The package contains several modules that implement the toolbox's functionality and the two sub-packages scot.backend and scot.builtin. The former contains backend modules that allow SCoT to use different implementations of low-level routines such as PCA, ICA, or model fitting. The other package implements built-in versions of these routines. See Figure 3 for an overview of the package structure.

SCoT depends on NumPy, SciPy (Oliphant, 2007), and optionally on scikit-learn (Pedregosa et al., 2011). Matplotlib is required for visualization only. Furthermore, SCoT contains the complementary eegtopo package, which is used for EEG topography plots based on spherical spline interpolation (Perrin et al., 1989).

Development is test-driven, with unit tests covering core functionality as far as the stochastic nature of the implemented algorithms allows. The source code is released under the open source MIT license and is available online at github.com/SCoT-dev/SCoT.

### 3.2. Backend mechanism

A design goal of SCoT is to keep external dependencies at a minimum. The backend mechanism provides a common interface that allows SCoT to optionally utilize routines from third-party software without introducing unnecessary dependencies. A backend consists of targets that represent thin wrapper functions, class implementations or even modules. These are stored in a global dictionary, where they can be accessed from within SCoT. The dictionary is updated when including a backend module. Thus, the user can easily select a backend by simply importing the module. SCoT comes with two backends: scot.backend.builtin, which provides basic implementations of backend functionality and scot.backend.sklearn, which provides wrappers for routines implemented in the external scikit-learn package.

The built-in ICA backend calls BINICA to perform Infomax ICA (Makeig et al., 1996). BINICA is an external binary, which is either shipped with EEGLAB (Delorme and Makeig, 2004), or separately available online^1^ and is downloaded on demand.

Currently, the following backend targets are used by SCoT: ICA (ICA function), PCA (PCA function), VAR (VAR model fitting class), and utils (miscellaneous helper routines). The backend mechanism relies on duck-typing, which allows users to easily create custom backends by simply putting their own functions in the global backend dictionary.

### 3.3. Workspace

The scot.ooapi module exposes the class Workspace, which provides convenient access to SCoT from interactive Python sessions. This class also serves as an example for usage of the more flexible low-level application programming interface (API), which is described in detail later. An instance of the Workspace class is optionally initialized with sampling rate, desired dimensionality reduction, number of fast Fourier transform (FFT) bins and/or electrode locations. This alleviates the user of the burden to pass these parameters to each individual function call. The Workspace class can perform source decomposition on EEG data, estimate connectivity on the same or a different data set, conduct statistical analysis, and visualize the results.

### 3.4. Package structure

Here, we provide a summary of the modules that form the SCoT package. Please refer to the API reference in the SCoT documentation for a more detailed description. A summary of the modules is listed in Table 2.

**Table 2:** **Python modules that form the SCoT API**.

**Module**	**Purpose**
backend	(sub-package) Backend interfaces
builtin	(sub-package) Implementation of the builtin backend
config	Global configuration
connectivity	Connectivity analysis
connectivity_statistics	Statistical evaluation of connectivity
datatools	Basic data manipulation
matfiles	Routines for loading and saving MATLAB. mat files
ooapi	Object oriented API (Workspace)
plainica	Source decomposition with ICA
plotting	Visualization
utils	Utility functions
VAR	VAR model interface
varica	Joint source/VAR estimation
xvschema	Cross-validation strategies

#### 3.4.1. Internal modules

These are modules mainly intended for internal use in SCoT.

builtin is actually a sub-package. It contains SCoT's own implementations of PCA, CSP, VAR, and a wrapper to the BINICA binary.

The config module defines global variables for configuring SCoT. Currently, it contains only the backend dictionary. This dictionary is populated when importing a backend. Functions like mvarica query this variable to determine which implementations of PCA, ICA, etc., to use.

Internal utility functions are defined in the utils module. These include among others a function for calculating autocovariance matrices and the memoize decorator that caches function return values.

#### 3.4.2. User facing modules

These are modules that users of SCoT will often work with.

backend is actually a sub-package. It contains a separate module for each backend currently available in SCoT. At the moment these are builtin and sklearn.

The connectivity module provides a class for extracting connectivity measures from VAR model coefficients (see Table 1 for a list of available connectivity measures). Some connectivity measures are calculated from other measures (e.g., the dDTF). To avoid unnecessary recalculations of repeatedly used measures, this class makes use of the memoize decorator to quickly get the cached return values of member functions that have been called before.

Functions for statistical evaluation of connectivity are available in connectivity_statistics. These include surrogate estimation under the null-hypothesis of no connectivity, bootstrapping, statistical tests, and correction for multiple testing.

The datatools module contains functions for manipulating EEG data, such as cutting segments from continuous data or applying spatial filters to segmented data. The matfiles module allows loading and saving of MATLAB.mat files. This module converts the result of scipy's loadmat to nested Python dictionaries.

The ooapi modules provides the Workspace class, which is described in detail above.

ICA source decomposition is implemented in the plainica module. It performs optional dimensionality reduction with PCA and subsequent ICA source decomposition.

The plotting module contains visualization routines. This module depends on matplotlib to create plots similar to MATLAB. The dependency is optional and required only for visualization; if matplotlib is not available, the module can still be imported, but the functions cannot be called. The visualization routines rely on the eegtopo package to plot scalp projections of sources.

The VAR module contains the class VARBase, which is the VAR base class for VAR models in SCoT. This class implements routines common to all implementations of VAR models, such as prediction or model validation. However, model fitting routines are provided by the derived classes scot.builtin.var.VAR and scot.backend.sklearn.VAR.

The MVARICA and CSPVARICA procedures are implemented in the varica module. The module exposes one function for each procedure.

Cross-validation strategies are implemented in xvschema. This module contains functions that generate indices for testing and training sets for single-trial and multi-trial optimization. While multi-trial strategy is a normal leave-one-out cross-validation, the single-trial strategy creates training sets that contain only single trials.

## 4. Results

### 4.1. Using SCoT

#### 4.1.1. Basic usage

Here, we will demonstrate how to use SCoT to estimate multi-trial connectivity from EEG data. More detailed examples are distributed with the source code.

First, the SCoT package must be made available to the Python interpreter. By default, SCoT uses the built-in routines for PCA and ICA. Alternatively, scikit-learn can be used by importing the sklearn backend.


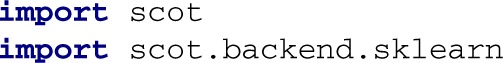


SCoT works with three-dimensional NumPy arrays. The three dimensions of an EEG data set are time, signals, and trials. An example data set is available with SCoT. This data set contains a recording of 45 EEG channels from one subject performing hand and foot MI. The subject was instructed to perform either MI task by a visual cue. Every 9.5–10.5 s such cues were presented 90 times for each class in randomized order.


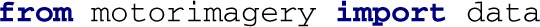


In the following code snippets we use the variables raweeg, triggers, classes, and fs. These variables are taken from the example data set and contain the continuous EEG data (samples × channels), a list of trigger locations (sample indices) that mark individual trials, class labels (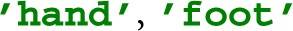) for each trial, and the sampling rate (Hz).

Convenience functions for basic data manipulation are available. The following example cuts segments of 1 s starting 3 s after each trigger from continuous EEG and arranges them in three dimensions as described above.


eeg =
  scot.datatools.cut_segments(
  raweeg, triggers, 3*fs, 4*fs)


The Workspace class provides a high-level interface to the toolbox. As the name suggests, an instance of this class provides a workspace on which SCoT routines operate. The workspace contains data, source and connectivity estimates, and settings.


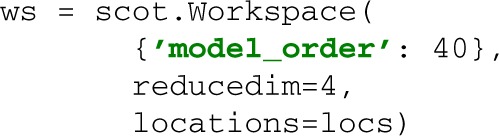


This command initializes a new workspace with VAR model order 40, dimensionality reduction to four components, and EEG electrode locations described in the variable locs.

If reducedim was not set, it would default to retaining 99 % of the EEG variance. Alternatively, PCA can be disabled by setting reducedim to 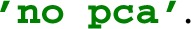.

A dataset is passed to the workspace with the set_data method. The optional second argument may contain a list of labels that assigns a class label to each trial in the data. The method do_mvarica decomposes the EEG data into source activations and fits a VAR model in the process. It is important to test the VAR residuals for whiteness. A *p*-value of less than e.g., 0.05 returned by VAR.test_whiteness would indicate significantly non-white residuals, and the VAR settings would need to be tuned. To obtain separate VAR models for each class, we call set_used_labels() to specify which classes to use in subsequent operations. Once VAR models are fitted with the fit_var method, we can plot spectral connectivity measures with get_connectivity. Finally, show_plots displays the plots.


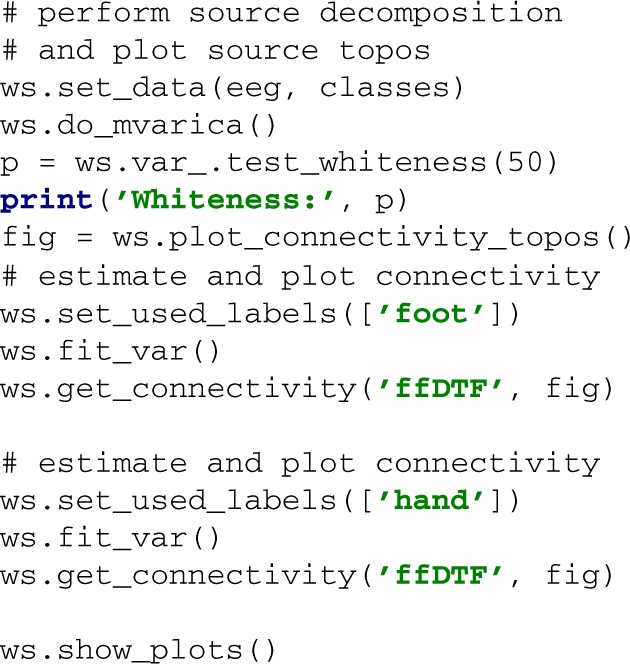


**Figure 4:**
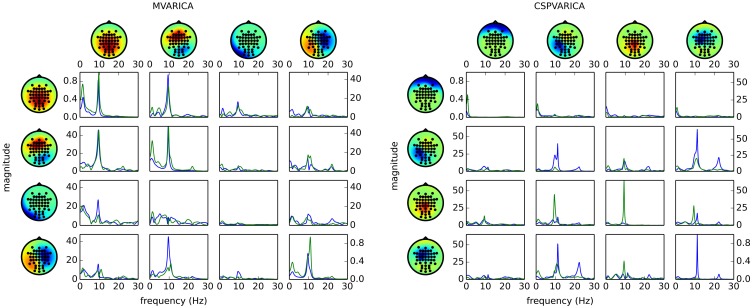
**Directed connectivity with MVARICA (left) and CSPVARICA (right)**. The ffDTF between four components is plotted for hand (green) and foot (blue) motor imagery. The estimation window was set from 3 s to 4 s after cue on-set. The *x*-axes correspond to frequencies from 0 to 30 Hz, and the *y*-axes are the magnitude of the ffDTF (in arbitrary units). Columns correspond to sources and rows to sinks. Scalp projections are arbitrarily scaled to provide a qualitative representation of the sources. Each source's power spectral density is plotted along the diagonal for reference.

Figure 4 (left) shows the result of applying these steps to the example data set. By replacing do_mvarica with do_cspvarica, we obtain different sources (Figure 4, right). The two frequency bands μ (8–12 Hz) and β (16–24 Hz) are known to play a part in motor processing (Pfurtscheller, 1981). While MVARICA detects connectivity mostly in the μ band, CSPVARICA reveals connectivity in the μ and β bands. Furthermore, connectivity between the CSPVARICA sources varies more between classes. This difference between MVARICA and CSPVARICA is somewhat expected, because MVARICA selects sources that explain as much of the EEG variance as possible, while CSPVARICA prefers sources with maximally different activations between classes.

#### 4.1.2. Time-varying connectivity

The plots in Figure 4 only show a snapshot of connectivity in one time segment. In order to get an overview on how connectivity evolves over time, the estimation process is repeated for multiple time-shifted segments. However, source decomposition should not be performed for every time segment individually. Even if the same sources were detected in consecutive windows, their signs and order would change randomly. This in turn would make interpreting the results very difficult. Instead, it is reasonable to re-use the same sources (represented by the unmixing matrix) in each time segment.

In the following example, such time-frequency analysis is performed over the whole trial of the data set. The new data set is prepared by cutting 10 s long slices starting 2 s before each trigger from the continuous EEG.


eeg_long =
  scot.datatools.cut_segments(
  raweeg, triggers, -2*fs, 8*fs)


The source decomposition obtained before from the short time segments will be re-used by simply assigning the new data set to the workspace. Time-frequency analysis does not require the user to call fit_var. Instead, separate models are fitted internally for each time segment. The function get_tf_connectivity takes three mandatory arguments: the measure to use, window length, and step size.


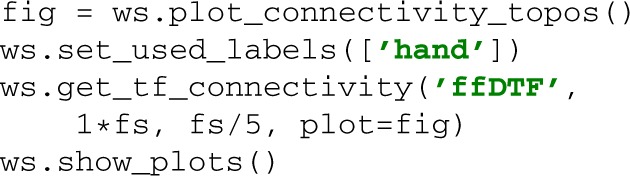


**Figure 5:**
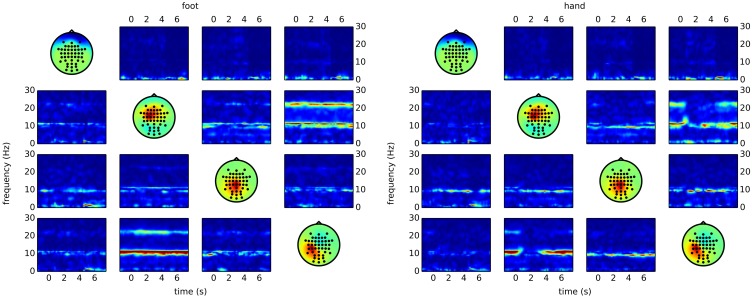
**Time/frequency plots as obtained with CSPVARICA**. The ffDTF between four components is plotted for foot (left) and hand (right) motor imagery. A 1 s long estimation window was sliding between *t* = −2 and *t* = 8. The *x*-axes correspond to the center of the sliding window which ranges from −1.5 to 7.5 s, where *t* = 0 represents the trigger (cue). The *y*-axes correspond to frequencies from 0 to 30 Hz. Columns correspond to sources and rows to sinks, while the diagonal shows the scalp projections of the respective components. Scalp projections are arbitrarily scaled to provide a qualitative representation of the sources.

The code snippet above produces output similar to the right side of Figure 5. Here, it becomes clear that most of the differences between the classes are due to reduced connectivity during hand motor imagery.

### 4.2. BCI simulation

Using SCoT, we performed a BCI simulation study to demonstrate the efficacy of CSPVARICA and MVARICA on single trial classification on EEG recordings of MI data. Fourteen subjects participated in this study, all of them gave informed consent and were paid for their participation. Each participant took part in two sessions on separate days, with six recording runs of 30 trials in each session. The sessions comprised 90 right hand MI and 90 foot MI trials. Trial duration was 7 s with breaks of varying duration (2.5–3.5 s) between trials. EEG preprocessing included removing electrooculogram (EOG) artifacts (Schlögl et al., 2007) and downsampling to 100 Hz. A more detailed description of the data and preprocessing procedure can be found in Billinger et al. (2013a).

We performed cross-validation per subject and session, using each of the six runs for testing once and the remaining five runs for initializing the procedure. In each cross-validation step, we decomposed the raw EEG into 16 components with either CSPVARICA or MVARICA. Subsequently, we split the component activation signals into segments of 1.5 s length that overlapped by 0.2 s. The following two steps were applied to each segment individually. First, we determined the optimal regularization parameter λ for subsequent single-trial VAR model fitting. Second, we extracted full frequency directed transfer function (ffDTF), ffPDC, and band power (BP) features in two frequency bands (7–13 Hz and 15–25 Hz). While ffDTF and ffPDC were based on the VAR model, logarithmic BP was calculated directly from the time signals to serve as a baseline. Finally, we trained a shrinkage linear discriminant analysis (sLDA) classifier on the time segment where classes were best discriminated for each feature type.

We tested the procedure on the run that was previously withheld from initialization. First, we decomposed the EEG into the same components as in the initialization step by spatially filtering the test set with the unmixing matrix obtained during initialization. For each time segment, we subsequently fitted regularized VAR models individually on every trial, extracted the ffDTF, ffPDC, and BP features, and applied the classifier. This resulted in a confusion matrix for each time segment. We calculated Cohen's kappa κ for each segment during the MI phase. The κ metric is preferable over classification accuray because it takes class distribution into account (Billinger et al., 2013b). From all segments, we took the 0.9 quantile of κ as classification performance, which is less sensitive to outliers than peak performance. This measure of classification performance was obtained for each subject and session.

**Figure 6:**
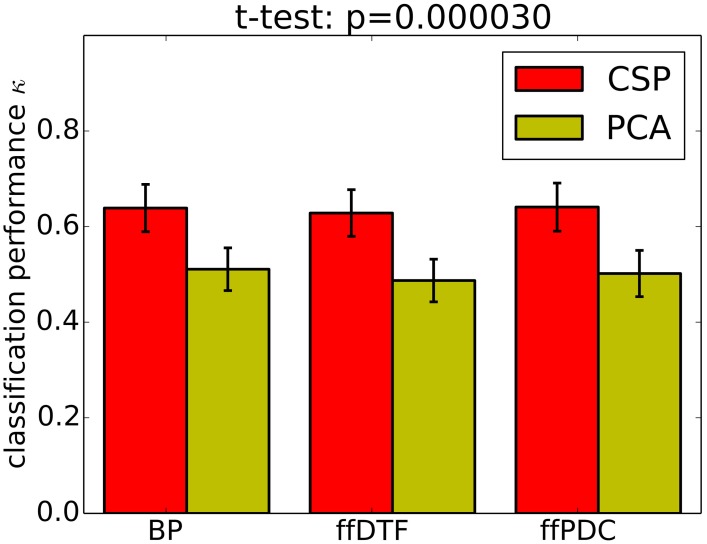
**Classification performance (0.9 quantile of Cohen's kappa) for PCA-based dimensionality reduction (MVARICA) and CSP-based dimensionality reduction (CSPVARICA)**. The standard error of mean classification performance is indicated on top of the bars.

Classification performance is significantly higher with CSPVARICA than with MVARICA (Figure 6). Thus, CSPVARICA seems to be preferable over MVARICA for MI classification. It is also reasonable to assume that CSPVARICA is useful for studying connectivity under varying conditions or tasks.

**Figure 7:**
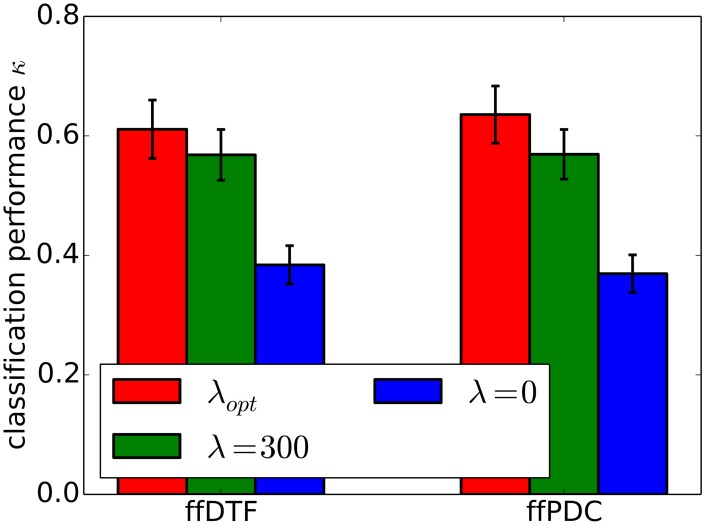
**Classification performance (0.9 quantile of Cohen's kappa) for different regularization approaches**. Regularization was optimized for individual subjects (λ_opt_), set to zero (λ_0_), and to a high value (λ_300_). The standard error of mean classification performance is indicated on top of the bars.

Furthermore, we demonstrate the effects of regularization on classification performance. In Figure 7, we compare per-subject optimization of the regularization parameter with no regularization and with applying over-regularization by setting the parameter to a high value. Optimal regularization was determined in the initialization phase of the cross-validation for each time segment individually, resulting in κ = 0.62 ± 0.18. Clearly, no regularization performs worst with κ = 0.38 ± 0.11. For over-regularization, we chose λ roughly an order of magnitude higher than the average optimal value, which slightly decreased classification performance to κ = 0.57 ± 0.15. However, the impact of too much regularization is difficult to quantify.

## 5. Discussion

In this article, we introduced SCoT, the Python toolbox for source connectivity estimation. It provides tools for ICA-based source decomposition, VAR model fitting, and extraction of connectivity measures.

Widely used neuroscience software packages^2^ (Hanke and Halchenko, 2011) such as EEGLAB (Delorme and Makeig, 2004), Fieldtrip (Oostenveld et al., 2011), and Biosig (Schlögl and Brunner, 2008) support connectivity analysis. Particularly, the SIFT toolobx (Delorme et al., 2011) includes routines for adaptive and segmented VAR model fitting with various smooth and sparse regularization techniques. Furthermore, SIFT supports SCSA (Haufe et al., 2010) for jointly estimating sources and connectivity. These packages are released under open source licenses and are available for MATLAB^3^. In contrast, the MNE software package^4^ supports model-based source reconstruction and exposes functionality for calculating several non-parametric connectivity measures in a Python API. With SCoT, we attempt to provide reusable and modular routines, which can help application developers avoid re-implementing the wheel in future projects based on Python.

For the first time, we presented our new CSPVARICA method and demonstrated that it is a useful source decomposition approach for data that contains different labeled conditions. Currently, our implementation supports only two conditions, but generalization to an arbitrary number of conditions is planned for a future release. Our BCI simulations showed that CSPVARICA outperforms MVARICA in terms of classification performance. This is not surprising since CSPVARICA favors sources that contain highly discriminative signals. However, MVARICA might be less susceptible to noise because it retains high-variance components. Whether or not one of these methods yields physiologically more meaningful results is an open question.

Although we conceived CSPVARICA mainly for application in BCIs, it is likely to be useful for other disciplines of neuroscience as well. Therefore, we encourage researchers to consider using CSPVARICA when analyzing differences between conditions.

SCoT relies on MVARICA or CSPVARICA for source decomposition. Alternative joint source/connectivity estimation techniques such as SCSA (Haufe et al., 2010) have not been implemented yet in SCoT. Furthermore, model-based source reconstruction is not included, because source localization is not within the scope of SCoT. However, SCoT can work with source decompositions obtained from such approaches by utilizing unmixing matrices obtained from other software packages.

VAR model fitting in SCoT is performed with regularized least squares optimization in general. Routines for ridge regression are built in, and other approaches such as Lasso, Elastic Net, LARS, or Bayesian regression are available through scikit-learn. Our simulations show an improvement in classification performance from κ = 0.38 without regularization to κ = 0.62 when applying ridge regression, which underscores the importance of regularization in single-trial connectivity. Support for more VAR fitting and regularization strategies is planned. A noteworthy approach is the group LASSO (Vidaurre et al., 2013), which promotes sparse connectivity. This could prove useful for the visualization of large networks, as it limits the number of non-zero connections.

Time-varying connectivity analysis is possible in SCoT by employing a sliding window. An alternative could be adaptive VAR models. However, adaptive models can be more difficult to handle due to their inherent exponential window. Furthermore, such models need to be updated for every single sample, while sliding windows can skip an arbitrary number of samples.

Our default implementation of ICA uses an external Linux binary to perform Infomax ICA. On other platforms, the FastICA implementation from scikit-learn may be used instead. Furthermore, the flexible backend mechanism should make it easy to include other ICA implementations such as CUDAICA (Raimondo et al., 2012) or the ICAs shipped with MDP (Zito et al., 2009).

In summary, SCoT provides tools required for estimating connectivity on EEG data to the free and open Python platform. It is designed to tightly integrate with popular scientific computation and visualization modules in order to be accessible to researchers familiar with Python.

### Conflict of interest statement

The authors declare that the research was conducted in the absence of any commercial or financial relationships that could be construed as a potential conflict of interest.

## Footnotes

^1^http://sccn.ucsd.edu/eeglab/binica/

^2^http://neuro.debian.net/survey/2011/results.html

^3^http://www.mathworks.com/products/matlab/

^4^http://www.martinos.org/mne/
